# State of the art on the main intervention methodologies in the field of emergency psychology: a systematic review

**DOI:** 10.3389/fpsyg.2025.1597897

**Published:** 2025-07-16

**Authors:** Michele Romanelli, Flavia Taddeo, Gian Piero Turchi, Antonio Iudici

**Affiliations:** ^1^Institute of Psychology and Psychotherapy, Milano, Padova, Italy; ^2^Department of Philosophy, Education, Sociology and Applied Psychology of Padua (FISPPA), University of Padua, Padua, Italy

**Keywords:** emergency psychology, catastrophe, crisis intervention, health promotion, vulnerability, systematic review

## Abstract

In recent years, emergency psychology has emerged as an interdisciplinary discipline that integrates clinical, community and intercultural approaches to managing the psychological impact of critical events. However, the rapid evolution of the field has generated methodological fragmentation, hindering the definition of a unified disciplinary identity. While international guidelines (IASC and OMS) promote an integrated approach, other models focus on PTSD prevention and practitioner training. This review analyses the main types of interventions in the literature through a systematic analysis and thematic clustering of 27 articles. The results highlight a wide range of approaches, from methodologies for the development of coping skills and social adaptation, to psychological support strategies, to clinical-diagnostic models borrowed from emergency medicine. However, the risk of reducing emergency psychology to an extension of the biomedical model, focused on the diagnosis and prevention of psychopathology, raises questions about the specificity and distinctive contribution of the discipline. The review underscores the need for a paradigm shift in emergency psychology toward more holistic, integrated, and community-centered approaches, emphasizing the importance of developing interventions that address both individual and collective resilience in crisis situations. The study’s scope was limited by its focus on English-language articles from the past decade and the use of specific keywords, potentially overlooking relevant interventions and alternative perspectives that could have emerged from a broader, multilingual search strategy. In terms of future research, this perspective suggests the need to develop methodologies and intervention protocols that go beyond clinical diagnosis and foster governance of interactions in emergency contexts, promoting effective and shared crisis management.

## 1 Introduction

Recent years have seen a significant increase in contributions in the field of humanitarian assistance and a gradual expansion in the implementation of psychosocial programs, which have highlighted the importance of psychological support in interventions aimed at assisting groups and communities affected by critical events (Di Maria, 2012).

These various experiences have undoubtedly enriched the landscape of interventions and strengthened local service networks, shaping what is now called emergency psychology (Farazmand, 2001). However, while emergency psychology has facilitated a range of activities and intervention projects in crisis situations and contexts, the rapid development of the field has also led to a significant broadening of its intervention focus. Today, contributions in the field qualify it as one of the most prolific disciplines. Over time, many authors and organizations have attempted to delineate the field of emergency psychology, resulting in a somewhat fragmented picture. For instance, the American Psychological Association (APA) sees emergency psychology as an interdisciplinary field with interventions aimed at addressing the immediate concerns of people affected by disasters and providing psychological support. According to the international guidelines from IASC (2006) and WHO (WHO, 2001), emergency psychology integrates traditional clinical psychology approaches with psychosocial, community, and cross-cultural dimensions of intervention.

The work of an emergency psychologist, therefore, is not limited solely to individual clinical support for victims of traumatic events but is also directed toward managing the psychosocial and community context in which the emergency occurs, attributing meaning to the event (Axia, 2006). The Réseau National d’Aide Psychologique d’Urgence (RNAPU) defines emergency psychology as the immediate and post-immediate psycho-social and spiritual support provided to disaster victims (Réseau National d’Aide Psychologique d’Urgence, 2022). Other authors (Alexander and Klein, 2009) have suggested that the psychological management of emergencies is crucial not only for the immediate support of victims but also for preventing long-term disorders such as PTSD (post-traumatic stress disorder). They describe emergency psychology as the discipline dealing with the psychological impact of critical incidents and disasters, with a particular focus on the prevention and management of psychological disorders. Jeffrey T. Mitchell’s (2016) Critical Incident Stress Management (CISM) model defines emergency psychology as a field concerned with the prevention and management of traumatic stress among those involved in critical emergencies. Finally, other definitions emphasize the importance of psychological preparedness and training for emergency responders, healthcare providers, and communities (Barbato et al., 2006).

Emergency psychology, being a multidisciplinary field, involves a network of professional figures with distinct yet strongly interconnected roles (Pietrantoni and Prati, 2009). Some psychologists are responsible for managing the psychological reactions of victims, family members, first responders, and affected communities, but they collaborate with emergency physicians and nurses who intervene in first aid or care for potential casualties. Furthermore, psychologists collaborate with civil protection operators who are responsible for coordinating logistics and security, collaborating with law enforcement agencies. Finally, psychologists work in conjunction with social workers who play a fundamental role in supporting post-emergency social and economic reintegration through the activation of territorial resources (housing, subsidies, family assistance) and institutions.

All of this highlights that emergency psychology is a field experiencing—and likely to continue experiencing—significant development and diversification, particularly at the methodological and operational levels. Each definition emphasizes different aspects of emergency psychology interventions, from clinical and community approaches to psycho-social and spiritual support, PTSD prevention, and strategic emergency preparedness and management. Given the rapidly evolving nature of the field, we question whether the complexity of interventions presents a unified or fragmented picture. We believe that the use of approaches that span different areas of interest and numerous fields of application could lead practitioners in this field to lack shared methodologies and practices, risking an overlap between emergency psychology interventions and other types of interventions (such as clinical, medical, or community-based interventions).

Therefore, in order to promote a distinctive placement of the main types of interventions within emergency psychology, this review aims to explore the following research questions: What are the types of interventions? What might be the unique or common elements among interventions? What is the direction and purpose of the interventions? And finally, what are the methodologies or strategies currently employed in various interventions?

The aim of this review is to describe the current state of the primary intervention methodologies in emergency psychology. The goal is to provide a generalized map of the various intervention proposals currently used within the discipline, which could be valuable for professionals in the field to assess how distinct the discipline can be in its proposed interventions.

## 2 Method and data

This research was conducted using the methods of systematic literature review (Flick, 2009; Aromataris and Pearson, 2014; Pati and Lorusso, 2018). The primary objective of a literature review is to conduct an in-depth and critical analysis of what has already been written and studied on a specific topic. We chose this method because we observed that publications in the field of emergency psychology over the last decade have developed significantly but across diverse themes. We thus felt the need to conduct a state-of-the-art review of existing works. This methodology was also selected to identify unexplored areas or unresolved issues in the field of study.

### 2.1 Data collection, inclusion criteria, and selection procedures

**Figure 1:**
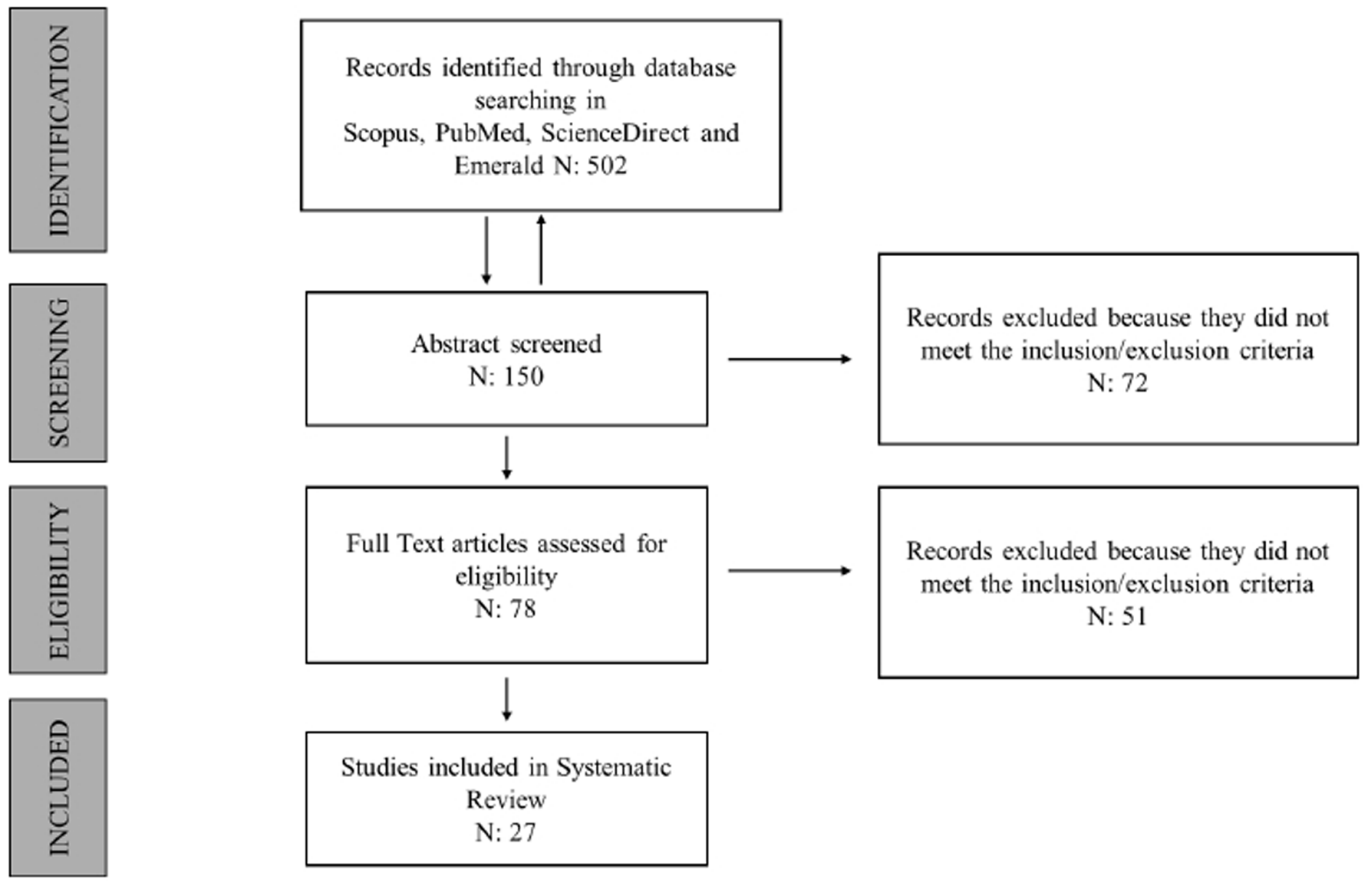
PRISMA flow diagram illustrating the processes of literature searches and screening.

The literature review was conducted using the following online databases: Scopus, ScienceDirect, and PubMed, searching for abstracts and content from 2014–2025 (see Figure 1). We followed procedures related to literature study, such as using general keywords, identifying a research topic (emergency psychology, intervention methodologies and strategies, psychological first aid, calamities, catastrophes, disasters, trauma, cataclysm), and defining conceptual maps (intersection circles between different themes and processes) within qualitative research.

The inclusion criteria were: (a) indexed articles or book chapters, (b) articles written in English, (c) journals, and (d) areas of application in which emergency psychology operates; (e) methodologies, approaches, and strategies used to manage an event where emergency psychology was employed, (f) articles citing emergency psychology in the period January 2024–January 2025; (g) the use of the following keywords (emergency psychology, intervention methodologies and strategies, psychological first aid, calamities, catastrophe, disaster, trauma, cataclysm).

The database search yielded a total of 854 articles. Of these, 332 were identified from ScienceDirect, 221 from Scopus, and 301 from PubMed. A second phase involved screening titles and abstracts. In this phase, 72 articles were excluded as they were not relevant to the field of emergency psychology. Abstracts of 782 articles were read, from which the most cited emergency areas were identified and considered as inclusion criterion (a): COVID-19; Psychological First Aid (PFA); pregnancy; terrorist attacks; earthquakes; resilience and post-traumatic stress disorder (PTSD). In a further screening step, 662 articles that did not meet inclusion criterion (a) were excluded. In the third phase, the remaining 120 articles that met inclusion criterion (a) were read in full. It emerged that only 27 fully complied with both inclusion criteria (a and b) Figure 1 illustrates this process.

### 2.2 Methodological quality assessment

The assessment tools used are the clarity of study objectives, the adequacy of study design, the validity of results, and the bibliographic value of the findings. Given the complexity of the topic, we divided the general theme into sub-concepts, called intersection areas. The authors of this work supervised the selection process, which took place in three distinct phases. First, articles were analyzed according to inclusion criteria; second, articles were evaluated based on title and abstract; and third, they were directly acquired.

As with any analytical method in qualitative research, document analysis required examining the data and interpreting it to obtain meaning and understanding, as well as to develop empirical knowledge (Rapley, 2011; Corbin and Strauss, 2008). The results of this review were reported in line with the Preferred Reporting Items for Systematic Review and Meta-Analysis (PRISMA) guidelines (Page et al., 2021). Based on the type of our work, the critical appraisal tool used in this research is the CASP (Critical Appraisal Skills CASP Systematic Review checklist, 2023). This checklist is based on several criteria including Focused Question, Right Papers, All Studies Included, Quality Assessed, Reasonable Combination, Overall Results, Precision, Local Application, Outcomes Considered, and Benefits Worth Costs (Table 1).

**Table 1:** Selection of studies.

	Title	Author	Country	Study proposal	Type of study and method
1	Psychological crisis and emergency intervention for frontline critical care workers during the COVID-19 pandemic	Gálvez-Herrer et al. (2021)	Spain	To explore the main feelings and coping strategies among frontline ICU physicians during the first phase of the COVID-19 pandemic and to assess the level of satisfaction after psychological crisis and emergency intervention	Psychological crisis care; content analysis; intervention satisfaction survey.
2	Should we offer disaster preparedness and response training workshops across Idaho? A feasibility study	Iqbal et al. (2022)	USA	To assess the impact of the workshop in improving participants’ knowledge and attitudes toward disaster management	Correlation analysis
3	The experience of the emergency psychologist during the COVID-19 pandemic	Rossi et al. (2021)	Italy	Exploring the psychologist’s experiences during COVID-19 emergency intervention.	Exploratory study; qualitative design; focus group method
4	Parent experiences and psychosocial support needs 6 months following paediatric critical injury: A qualitative study	Foster et al. (2019)	Australia	To explore parents’ experiences and needs for psychosocial support in the 6 months following the child’s serious injury.	Qualitative design; semi-structured interviews; thematic analysis
5	Building resilience for healthcare professionals working in an Italian red zone during the COVID-19 outbreak: A pilot study	Giordano et al. (2022)	Italy	Evaluate the impact of the R2 resilience program tailored for health leaders working in an area highly affected by COVID-19.	Quasi-experimental pre-test/post-test design; resilience-oriented multisystem approach
6	Empowering local response and community-based disaster mitigation through legislative policies	Hakkim and Deb (2023)	India	Highlight the critical role of local volunteers in disaster mitigation and preparedness, community response, and provide a framework and actionable measures to concretize the call for disaster risk reduction practices.	Qualitative studies
7	People’s experiences of distress and psychosocial care following a terrorist attack: Interviews with survivors of the Manchester arena bombing in 2017	Stancombe et al. (2022)	UK	Enhance understanding of the experience of distress among people attending the 2017 Manchester Arena bombing, identify their experiences of psychosocial care after the incident, and learn how to deliver and target effective psychosocial care after major incidents.	Thematic analysis; semi-structured interviews
8	Integrating mental health and disaster preparedness in intervention: a randomized controlled trial with earthquake and flood-affected communities in Haiti.	James et al. (2020)	USA	Examine the association between disaster exposure, disaster preparedness, and mental health symptoms.	Correlational research, mediation models,
9	Social and occupational factors associated with psychological distress and disorder among disaster responders: a systematic review	Brooks et al. (2016)	UK	Conduct a systematic literature review to identify social and professional factors that influence the psychological impact of disasters on responders.	interviews
10	Impact of the Las Vegas Mass Shooting Event on the Graduate Medical Education Mission: Can There Be Growth from Tragedy?	Guldner et al. (2023)	USA	Determine the psychological impact of the 2017 Las Vegas mass shooting on the university medical education mission (GME)	Univariate associations between continuous variables and correlational research
11	Post-traumatic stress disorder among civilians 6 and 18 months after the January 2015 terrorist attacks in the Paris region	Vincent et al. (2023)	Francia	To identify factors associated with medium- and long-term PTSD among people exposed to a terrorist attack in France	IMPACTS survey; relationships between latent variables
12	Risk factors for long-term post-traumatic stress disorder among medical rescue workers appointed to the 2008 Wenchuan earthquake response in China	Schenk et al. (2017)	China	To determine the risk factors for post-traumatic stress disorder (PTSD) among Chinese medical responders one year after the response to the Wenchuan earthquake on May 12, 2008	Impact of Events Scale-Revised (IES-R); univariate and multivariate analysis
13	Posttraumatic growth among health care workers on the frontlines of the COVID-19 pandemic	Feingold et al. (2022)	USA	To examine the prevalence, determinants or correlates of post-traumatic growth (PTG) among frontline health workers	Multivariate logistic regression analysis
14	Prevalence and factors of posttraumatic growth among Hubei residents during the COVID-19 pandemic: A cross-sectional study	Liu et al. (2024)	China	To investigate the prevalence and factors of post-traumatic growth (PTG) among residents in the most affected areas of China (Hubei province)	Correlational analysis and multiple linear regression
15	Healthcare utilization after mass trauma: a register-based study of consultations with primary care and mental health services in survivors of terrorism	Stene et al. (2022)	Norway	To investigate how the use of primary care physicians (PCPs) and mental health services (MHS) by young survivors changed after a terrorist attack.	Qualitative research
16	The need for additional mental health support for women in the postpartum period in the times of epidemic crisis.	Chrzan-Dętkoś et al. (2021)	Poland	To identify a possible intensification of mental health difficulties among women who sought support in the postpartum period during the epidemic state in Poland.	Screening with the Edinburgh Postnatal Depression Scale (EPDS)
17	Psychological First Aid: A Model for Disaster Psychosocial Support for the Perinatal Population	Giarratano et al. (2019)	USA	Demonstrate ways in which nurses can integrate psychosocial and interpersonal interventions into perinatal disaster care using Psychological First Aid (PFA)	Searching for articles
18	Provision of mental health services immediately following a natural disaster: Experiences after Hurricane Maria in Puerto Rico	Alto et al. (2021)	Porto Rico	Identify strategies for providing mental health services immediately after a natural disaster.	Qualitative research; thematic analysis; focus group method
19	Psychological First Aid for Wilderness Trauma: Interventions for Expedition or Search and Rescue Team Members	Mortimer and Mortimer (2023)	USA	Describe the preventive effect of PFA on the subsequent development of PTSD.	Research of articles
20	Psychological First-Aid Experiences of Disaster Health Care Workers: A Qualitative Analysis	Choi (2020)	South Korea	To examine how health workers experience mental health in disaster.	Qualitative research; phenomenological approach
21	The ABCDE psychological first aid intervention decreases early PTSD symptoms but does not prevent it: Results of a randomized-controlled trial	Figueroa et al. (2022)	Chile	Evaluate the effectiveness of the original PFA protocol, PFA-ABCDE, in preventing PTSD 1 month after the intervention and in reducing PTSD symptoms at one- and six-months follow-up.	Correlational study; SPSS; Composite International Diagnostic Interview (CIDI); Posttraumatic Checklist (PCL); Beck Depression Inventory-II (BDI-II)
22	Psychological First Aid Training: A Scoping Review of Its Application, Outcomes and Implementation.	Wang et al. (2021)	China	Investigate how PFA skills support people in acute distress, thereby improving self-efficacy and promoting resilience	Review by database search and literature search
23	The Association Between Disaster Vulnerability and Post-disaster Psychosocial Service Delivery Across Europe	Dückers et al. (2017)	Netherlands	Determine whether systems for planning and delivering post-disaster psychosocial support vary across Europe and identify elements that can guide improvement planning	Multilevel analysis; SPSS
24	Measuring and modelling the quality of 40 post-disaster mental health and psychosocial support programs	Dückers et al. (2018)	Netherlands	Measure quality domains recognized as relevant in the literature and empirically test associations	Survey; MHPSS program
25	Mental health workers perceptions of disaster response in China	Xi et al. (2019)	China	Provide recommendations for the development of a national mental health disaster response management plan in China	Qualitative study; focus group method; interviews
26	The need to introduce a psychological program into emergency medicine: Early experiences in the field	Landa-Ramírez and Murillo-Cruz (2019)	Mexico	Discuss the challenge of integrating psychology in the context of emergency medicine at the research and clinical levels	Search for articles;
27	A Systems Approach to International Disaster Psychology, Journal of Family Psychotherapy	Thoburn et al. (2014)	USA	Describe the history of international disaster psychology and outline contemporary theories and approaches that dominate the field	Biopsychosocial model

## 3 Findings

Based on the review of literature from the past 10 years, five distinct clusters were identified, within which the specific operational contributions of the interventions were detailed according to the particular methodologies and strategies employed. Using the criteria for assessing methodological quality and risk of bias based on the CASP list (Critical Appraisal Skills Programme, 2023) the results are categorized as high quality, moderate quality, and low methodological quality. Our work achieved 41% high quality, 45% moderate quality, and 15% low quality. These values are therefore considered to be of significant quality overall.

### 3.1 Methodologies for developing coping skills/“beyond individual resilience: integrated strategies for enhancing coping skills in emergencies”

The balance between job demands and the resources needed to effectively address them has emerged as a key factor in preventing mental health problems among healthcare workers during the COVID-19 pandemic. This approach, based on the Job Demands-Resource model, was explored in detail by Gálvez-Herrer et al. (2021) in the Spanish context. Their research highlighted how this balance can prevent issues such as burnout, anxiety, and depression, which were particularly prevalent among healthcare workers during the crisis. This study underscores the importance of organizational factors in individual coping, suggesting that effective coping strategies must be supported by appropriate workplace policies and resources, also in mental health (Iudici et al., 2022).

Practical training in disaster preparedness skills has proven particularly effective in improving operators’ perception of self-efficacy. This aspect was highlighted by Iqbal et al. (2022) through workshops conducted at Idaho State University. Their study demonstrated tangible improvements in participants’ knowledge and perception of self-efficacy, emphasizing the importance of integrating practical exercises into emergency operator training. This research points to the value of experiential learning in developing coping skills, suggesting that theoretical knowledge alone may be insufficient in preparing individuals for the realities of emergency situations.

The value of peer support and sharing experiences in structured contexts was underlined by Rossi et al. (2021), who analyzed the effectiveness of focus groups in the “Pronto Psy – COVID-19” service in Italy. Their research revealed how sharing experiences in a structured context can not only alleviate individual stress but also contribute to the development of collective coping strategies. This finding highlights the social dimension of coping, suggesting that effective coping strategies often emerge through collaborative processes rather than individual efforts alone.

A more holistic approach to coping, which includes the entire family unit, was proposed by Foster et al. (2019) in the context of pediatric emergencies. Their study highlighted the importance of cognitive-behavioral strategies that involve the whole family, suggesting that coping strategies in emergencies should be conceived more broadly, considering the individual’s social context. This research expands the scope of coping interventions beyond the individual, recognizing the interconnected nature of stress and resilience within family systems.

The collective findings from these studies point toward a more comprehensive and nuanced understanding of coping skill development in emergency situations. They suggest a move away from purely individualistic models of coping toward more contextual, social, and systemic approaches. This evolution in thinking recognizes that effective coping is not just about individual psychological resources, but also about the broader social, organizational, and familial contexts in which individuals operate.

Moreover, these methodologies highlight the importance of proactive skill development and preparation, rather than reactive responses to stress. They suggest that building coping skills should be an ongoing process, integrated into professional training and organizational practices, rather than a last-minute intervention during crises.

The emphasis on practical skills and experiential learning, as seen in the Idaho State University study, represents an important development. It recognizes that coping in emergency situations often requires quick, intuitive responses that are best developed through hands-on experience and practice.

The focus on peer support and collective coping strategies, highlighted in the Italian study, also offers valuable insights. It suggests that creating opportunities for shared reflection and mutual support can be a powerful tool in developing resilience, both at individual and group levels.

Finally, the holistic, family-centered approach proposed in the pediatric emergency context opens up new avenues for intervention. It suggests that effective coping strategies must consider the broader social systems in which individuals are embedded, recognizing that stress and resilience often have ripple effects beyond the individual.

In conclusion, these methodologies for developing coping skills reflect a growing recognition of the complex, multifaceted nature of resilience in emergency situations. They point toward more holistic, context-sensitive, and proactive approaches that have the potential to significantly improve both individual and collective capacity to handle crises. The challenge moving forward will be to integrate these diverse insights into comprehensive training programs and organizational practices that can effectively prepare individuals and systems for the unpredictable challenges of emergency situations.

### 3.2 Methodologies for developing social adaptation/from leadership to community: a new paradigm for social adaptation in crisis situations

The resilience of healthcare leaders has emerged as a crucial factor in post-emergency social adaptation. The “R2” model, studied by Giordano et al. (2022), identified key protective factors such as interpersonal relationships, optimism, and organizational flexibility. This innovative study suggests that improving leaders’ resilience can have a cascading effect on the entire healthcare system, reducing perceived stress and burnout symptoms at all levels. The implications of this research are far-reaching, as it shifts the focus from individual resilience to systemic resilience, recognizing the pivotal role that leaders play in shaping organizational responses to crises.

At the community level, the fundamental role of local volunteering and the importance of integrating civil society into disaster response systems were highlighted by Hakkim and Deb (2023) in their study on the response to floods in Kerala. Their research challenges the traditional top-down approach to emergency management, emphasizing the importance of decentralizing power to local entities to promote more resilient communities. This paradigm shift toward community-led disaster response represents a significant evolution in emergency management thinking. It acknowledges the unique insights and capabilities that local communities possess, which are often overlooked in centralized response models.

The convergence of these two perspectives – leadership resilience and community empowerment – points toward a more holistic and adaptive approach to social adaptation in emergencies. It suggests that effective emergency response systems should be designed to harness both top-down leadership capabilities and bottom-up community engagement. This dual approach could potentially lead to more robust and flexible emergency response mechanisms that are better equipped to handle the complexities and uncertainties of diverse crisis situations.

Furthermore, the emphasis on social adaptation highlights the need for long-term thinking in emergency preparedness. Rather than focusing solely on immediate response, these methodologies encourage the development of sustained resilience at both individual and community levels. This approach could potentially reduce the long-term psychological and social impacts of emergencies, leading to faster recovery and increased preparedness for future crises.

### 3.3 Methodologies for psychological support and assistance/healing together: the evolution of psychological support from individual intervention to collective process

Preventive interventions aimed at reducing negative self-evaluations of victims and the therapeutic value of survivor groups were explored by Stancombe et al. (2022) in the context of the 2017 Manchester attack. Their study highlighted how participation in survivor groups can have long-term beneficial effects, representing a shift from more traditional intervention models. This research underscores the importance of peer support and shared experiences in the recovery process, suggesting that psychological interventions should not only focus on individual therapy but also on creating supportive communities among survivors.

An integrated approach to disaster preparedness, combining emotional support and practical skills, was proposed and tested by James et al. (2020) in Haiti. Their method, which integrates facilitated discussions, sharing of personal experiences, and practical training, has shown promising results in terms of social cohesion and increased disaster preparedness behaviors. This multifaceted approach recognizes that effective psychological support in emergencies goes beyond addressing immediate emotional needs and includes building practical resilience skills.

The importance of considering pre-, peri-, and post-disaster factors in the psychological impact on rescuers was emphasized by Brooks et al. (2016). Their research also highlighted the effectiveness of group psychological support in all phases of the emergency, underscoring the importance of a continuous and adaptable approach to psychological support in crisis situations. This longitudinal perspective on psychological support is crucial, as it acknowledges that the mental health impacts of emergencies can evolve over time and require ongoing, flexible interventions.

The collective findings from these studies point toward a more comprehensive and nuanced understanding of psychological support in emergencies. They suggest a move away from a one-size-fits-all approach toward more tailored, community-oriented, and phase-specific interventions. This evolution in thinking recognizes that psychological support should be an integral part of all stages of emergency management, from preparedness through to long-term recovery.

Moreover, these methodologies highlight the interconnectedness of individual and collective psychological well-being in crisis situations. They suggest that effective psychological support should aim to strengthen both individual coping mechanisms and community resilience. This dual focus could potentially lead to more sustainable outcomes, as it addresses both immediate psychological needs and builds long-term community capacity to handle future crises.

The emphasis on practical skills training alongside emotional support, as seen in the Haiti study, also represents an important development. It recognizes that psychological well-being in emergencies is closely tied to an individual’s sense of agency and ability to take concrete actions. This approach could potentially reduce feelings of helplessness often associated with traumatic events and empower individuals to play an active role in their own recovery and that of their communities.

In conclusion, these methodologies for psychological support and assistance reflect a growing recognition of the complex, multifaceted nature of mental health in emergency situations. They point toward more holistic, community-oriented, and empowering approaches that have the potential to significantly improve both immediate response and long-term recovery outcomes.

### 3.4 Methodologies for clinical diagnosis/diagnosis and growth: rethinking mental health in emergencies beyond pathology

The resilience of doctors in mass emergency situations and the risk of PTSD development in a minority of operators were examined by Guldner et al. (2023). Their study revealed that while most doctors manage to handle traumatized patients without experiencing significant trauma, a minority may develop PTSD symptoms, highlighting the need for a differentiated approach to psychological support. This research underscores the importance of recognizing healthcare providers not just as caregivers but also as potential victims of secondary trauma, necessitating tailored support systems within healthcare institutions.

The crucial importance of social support in preventing PTSD, anxiety, and depression among civilians affected by terrorist attacks was confirmed by Vincent et al. (2023). Their results suggest that post-disaster interventions should focus not only on clinical treatment but also on strengthening social support networks. This finding highlights the need for a more holistic approach to mental health in emergency situations, one that considers the social ecology of trauma and recovery.

Specific risk factors for PTSD development among healthcare workers were addressed by Schenk et al. (2017) in the context of the 2008 Wenchuan earthquake. Their proposal to integrate these factors into rescue programs represents a step forward toward more targeted and personalized interventions. This research points to the potential of predictive models in emergency mental health, which could allow for more proactive and preventive approaches to psychological support.

Post-traumatic growth (PTG) and factors associated with it, such as spiritual support and being healthcare workers, were explored by Feingold et al. (2022) and Liu et al. (2024). These studies have opened new perspectives on positive psychological responses to emergencies, suggesting that emergency response should consider not only the prevention of psychological disorders but also the promotion of personal growth. This shift toward a more positive psychology framework in emergency situations represents a significant evolution in the field, moving beyond a deficit model to one that recognizes the potential for positive transformation through adversity.

The debate on the effectiveness of Psychological First Aid (PFA) remains open. While some studies support its effectiveness (Giarratano et al., 2019; Alto et al., 2021), others raise doubts about its preventive function and the methodological rigor of evaluations (Figueroa et al., 2022; Wang et al., 2021). This controversy highlights the need for further research and more rigorous evaluation of psychological interventions in emergency situations (Iudici et al., 2022). It also underscores the challenges of conducting robust research in contexts and the importance of developing more sophisticated methodologies for assessing the impact of psychological interventions in real-world emergency situations.

The collective findings from these studies point toward a more nuanced and personalized approach to clinical diagnosis and intervention in emergency situations. They suggest a move away from one-size-fits-all approaches toward more targeted, context-specific interventions that consider individual differences, social factors, and the potential for both negative and positive outcomes.

Moreover, these methodologies highlight the importance of longitudinal studies in understanding the long-term impacts of emergencies on mental health. They suggest that clinical approaches should be flexible and adaptable, capable of addressing both immediate trauma responses and long-term psychological trajectories, including the possibility of post-traumatic growth.

The emerging focus on resilience and growth, alongside the traditional emphasis on diagnosing and treating psychopathology, represents a significant shift in the field of emergency mental health. This dual focus could potentially lead to more comprehensive and effective interventions that not only mitigate negative outcomes but also foster positive adaptation and personal development in the face of adversity.

### 3.5 Methodologies for building networks among local services/weaving the resilience network: building integrated response systems for future emergencies

The need for an integrated approach to post-disaster psychosocial support and the importance of a structured network of coordination between professionals, volunteers, and authorities were highlighted by Dückers et al. (2017, 2018). Their research emphasized that interdisciplinary collaboration is fundamental for an effective response to emergencies. This work underscores the complexity of emergency response systems and the need for well-coordinated, multi-stakeholder approaches that can effectively mobilize diverse resources and expertise.

A mental health crisis intervention system based on integrated teams and unified management was proposed by Xi et al. (2019) in the Chinese context. This approach reflects a global trend toward greater integration of mental health services in emergency responses. It highlights the growing recognition of mental health as a critical component of comprehensive emergency management, rather than an afterthought or separate concern.

The integration of psychology into emergency medicine was advocated by Landa-Ramírez and Murillo-Cruz (2019). Their work highlights how the psychological dimension is essential in patient care in emergency situations, on par with more traditional medical considerations. This perspective challenges the historical separation between physical and mental health care in emergency settings and points toward a more holistic, patient-centered approach to emergency medicine.

A paradigm shift toward a community-based approach, combining clinical management of PTSD, access to interpersonal resources, and family support, was identified by Thoburn et al. (2014). Their proposal represents a more holistic and integrated approach to mental health in emergencies, highlighting a shift from a clinical-pathological approach to a community-based one. This shift recognizes the importance of social and community factors in mental health outcomes and suggests that effective interventions must engage with broader social systems, not just individual patients.

The collective findings from these studies point toward a more comprehensive and integrated approach to building networks among local services in emergency situations. They suggest a move away from siloed, discipline-specific responses toward more collaborative, interdisciplinary approaches that can address the complex, multifaceted nature of emergencies.

Moreover, these methodologies highlight the importance of local context and community engagement in emergency response. They suggest that effective network-building should not only focus on formal institutions and professional services but also incorporate local knowledge, community resources, and informal support systems.

The emphasis on unified management and integrated teams, as seen in the Chinese study, also represents an important development. It recognizes the need for clear leadership and coordination in complex emergency situations, while also acknowledging the diverse expertise required for effective response.

In conclusion, these methodologies for building networks among local services reflect a growing recognition of the interconnected nature of emergency response systems. They point toward more collaborative, community-oriented, and integrated approaches that have the potential to significantly improve both the efficiency and effectiveness of emergency response efforts. The challenge moving forward will be to implement these insights in diverse contexts, navigating local cultural, political, and institutional landscapes to create truly responsive and resilient emergency management systems (Table 2).

**Table 2:** Synoptic overview of clustering.

**Cluster**	**Cluster description**	**Studies**
1 Methodologies for the development of coping skills	Interventions that consider the ways in which individuals cope with (or minimize) emergency situations.	Gálvez-Herrer et al. (2021), Iqbal et al. (2022), Rossi et al. (2021), and Foster et al. (2019)
2 Methodologies for the development of social adaptation	Interventions that consider the construct of resilience; that is, the “ability to adapt positively to adversity as a key factor in post-emergency recovery.”	Giordano et al. (2022) and Hakkim and Deb (2023)
3 Methodologies for psychological support and advocacy	Interventions that are characterized as psychological support and advocacy to mitigate the post-disaster psychological impact.	Stancombe et al. (2022), James et al. (2020), and Brooks et al. (2016)
4 Methodologies for clinical diagnosis	Diagnostic interventions aimed at defining post-disaster distress through labels from clinical psychology and the medical operating model	Guldner et al. (2023), Vincent et al. (2023), Schenk et al. (2017), Feingold et al. (2022), Liu et al. (2024), Stene et al. (2022), Chrzan-Dętkoś et al. (2021), Giarratano et al. (2019), Alto et al. (2021), Mortimer and Mortimer (2023), Choi (2020), Figueroa et al. (2022), and Wang et al. (2021)
5 Methodologies for building the network between area services	Interventions aimed at fostering networking among area services through guidelines and trainings aimed at experts, volunteers, clinicians, and other community roles	Dückers et al. (2017), Dückers et al. (2018), Xi et al. (2019), Landa-Ramírez and Murillo-Cruz (2019) and Thoburn et al. (2014)

## 4 Discussion and conclusion

Over the past decade, research in the field of emergency psychology has made significant strides, offering a rich and stimulating overview of intervention methodologies employed in crisis situations. A comprehensive review of the literature strongly indicates the necessity for a paradigm shift in emergency management (Dückers et al., 2017).

We can no longer afford to focus exclusively on the individual. Our perspective must broaden to consider the person within their wider social, organizational, and familial context. This shift in perspective has profound implications for practitioners in the field and opens new avenues for future research (Foster et al., 2019).

The organization of various contributions into clusters has proven to be a valuable tool. It enables professionals to systematize different intervention proposals with greater precision, highlighting both common and distinctive elements of each approach. From this analysis, three main areas of intervention emerge that warrant our attention.

Firstly, there is a clear need for a holistic and integrated approach. It is no longer sufficient to intervene solely on the individual level; rather, it is necessary to actively involve the entire social support network, including significant others. Promoting genuine interdisciplinary collaboration among various professionals - psychologists, physicians, social workers, and others–is fundamental (Gálvez-Herrer et al., 2021).

A second crucial aspect is the valorization of community resources. Research demonstrates the importance of enhancing and leveraging existing resources within local communities. Practitioners must learn to facilitate active involvement of civil society and local volunteers in emergency response (Hakkim and Deb, 2023).

The third key point concerns proactive preparation and long-term support. We can no longer afford to merely react to crises; we must be prepared to address them. This necessitates integrating practical exercises and simulations into practitioner training to develop concrete crisis management skills (Iqbal et al., 2022).

An interesting finding that emerges from the analysis is that approximately 30% of the selected studies refer to interventions that combine diverse methodologies, ranging from a focus on coping to social adaptation. These approaches aim to promote effective behaviors for managing or addressing problematic situations, working on both individual and group resources (Rossi et al., 2021).

However, there is an aspect that warrants particular attention: about 50% of the selected contributions belong to the cluster of Clinical Diagnosis Methodologies. Here, we observe a distinctive use of the medical operational model, with psychology assuming a supportive role, adopting a clinical-diagnostic approach to analyze post-disaster distress (Guldner et al., 2023).

In light of these results, a concrete risk emerges for emergency psychologists: that of limiting their contribution to the practices of the medical operational model, focusing on diagnosis from a preventive psychopathology perspective, rather than addressing the interactive modalities that define the emergency context and characterize a broader dimension of health (Schenk et al., 2017; Neri et al., 2020).

In this context, it could be particularly significant for the field of emergency psychology to adopt a broader definition of health, one that considers both the “medical” and “social” dimensions in interaction with each other (Turchi et al., 2019; Turchi et al., 2022), thus embracing a dialogic vision.

Looking to the future, crucial questions emerge for research and practice in emergency psychology: What distinctive contribution can this discipline offer? What methodologies and intervention protocols can it employ to establish itself as an independent discipline capable of managing complex interactions in emergency contexts? (Landa-Ramírez and Murillo-Cruz, 2019).

In this regard, it is advisable to organize conferences aimed at unitarily elaborating the potential of the discipline, reducing the risk of disarticulated development.

Future research will need to address several challenges: evaluating the real effectiveness of interventions such as Psychological First Aid, overcoming current methodological controversies (Figueroa et al., 2022); developing more sophisticated research protocols and conducting longitudinal studies to assess the long-term effects of interventions (Brooks et al., 2016); exploring themes of organizational resilience and leadership effectiveness in crisis situations (Giordano et al., 2022); studying operational models of collaboration between healthcare personnel and psychologists in emergency contexts (Xi et al., 2019); and analyzing the efficacy and implementation modalities of community-based approaches in emergency management (Thoburn et al., 2014).

In conclusion, we are faced with a complex and continuously evolving landscape of intervention methodologies in emergency psychology. The emerging operational implications necessitate a significant paradigm shift in the training and practice of practitioners, pushing us toward more holistic, integrated, and community-centered approaches. Future research will have the fundamental task of providing further evidence to guide this transformation process, with the ultimate goal of developing more effective, resilient, and adaptive emergency response systems.

### 4.1 Limitations

Finally, in evaluating the results obtained, some limitations are highlighted, which are useful for placing the interpretation of the results and considering possible alternatives for future research. The first limitation concerns the methods and data collection. A time frame was used that was restricted to the last ten years and only articles written in English. A proposal for future research could be to extend the research to include foreign language studies. A second limitation concerns the interpretation and discussion of the results, which is closely linked to the use of keywords in the article search and their selection. It is anticipated that by including alternative keywords (for example, more focused on the interventionist aspect of emergency psychology), studies could have been collected that might show other types of interventions. This last point leads to a third limitation regarding the clustering process: it is anticipated that with a broader number of articles, more focused on the intervention aspect of the discipline (through an expansion of the keywords), different clusters could have been identified, from which other elements could have emerged for discussion of the results.
